# Do Not “Let Them Eat Cake”: Correlation of Food-Consumption Patterns among Rural Primary School Children from Welfare and Non-Welfare Households

**DOI:** 10.3390/ijerph14010026

**Published:** 2016-12-28

**Authors:** Daniel Terry, Kaye Ervin, Erin Soutter, Renata Spiller, Nicole Dalle Nogare, Andrew John Hamilton

**Affiliations:** 1 Department of Rural Health, The University of Melbourne, Shepparton 3630, Australia; d.terry@unimelb.edu.au (D.T.); andrewjh@unimelb.edu.au (A.J.H.); 2 Goulburn Valley Primary Care Partnership, Shepparton 3630, Australia; souttere@ndhs.org.au (E.S.); hp2@gvpcp.org.au (R.S.); hp@gvpcp.org.au (N.D.N.); 3 Office of the Deputy Vice-Chancellor (Research and Innovation), Federation University Australia, Ballarat 3353, Australia

**Keywords:** food, drinking, healthy eating, eating behaviour, socioeconomic factors

## Abstract

Physical and financial access impacts food choice and consumption, while educational attainment, employment, income, gender, and socioeconomic status are also influential. Within this context, the aim of the paper is to examine the association between various foods consumed and eating patterns of children between low and higher income households. A paper-based survey was completed by parents/carers of children in 41 primary schools in rural and regional areas of Victoria. Data collected included demographics and the consumption of fruit, vegetable, and other foods including drinks. Ordinal data were analysed using Spearman’s rank-order correlation. The main findings were that children who consumed more fruit and vegetables tended to have a higher intake of healthy drinks (plain milk and water) as well as a lower intake of unhealthy snacks and drinks (sugar sweetened drinks). Those who perceived that fruit and vegetables cost too much reported greater consumption of unhealthy snacks and sugar-sweetened beverages, which was more prominent in low-income households. Changing food consumption behaviours requires a complex systems-based approach that addresses more than just individual issues variables. A participatory approach that works with local communities and seeks to build an understanding of unique challenges within sub-groups has potential for embedding long-lasting and meaningful change in eating behaviours.

## 1. Introduction

It has been established that low consumption of nutrient-rich foods, such as fruits and vegetables, has short- and long-term negative health implications [1]. Poor nutrition is responsible for greater morbidity and mortality than alcohol, tobacco, and low-level physical activity combined [2]. A number of dietary risk factors have been implicated in chronic conditions, such as cardiovascular disease, type 2 diabetes, and some cancers [3]. Low consumption of fruits and vegetables is more prevalent in low-socioeconomic populations [4]. Physical and financial access to food has been clearly shown to affect food consumption [5]. Food literacy, or knowledge of healthy eating, may also affect food choices and methods of preparation and consumption [5]. Other social determinants, such as educational attainment, employment, income, gender, and socioeconomic status, also influence food choice and food consumption [6,7].

Social cognitive theory recognises that individual, social, and environmental factors contribute to the complexities of food consumption [4,7]. Due to individual influences, such as beliefs, perceptions, and values, what is considered “unhealthy” within one group, may be considered “healthy” by another group [7]. For example, higher socioeconomic individuals are often more concerned with health and body image, which affects food consumption. Conversely, lower socioeconomic individuals may be more accepting of being overweight, and being overweight may in some cases be considered desirable [4,7]. However, whilst being overweight may be more common among low socio-economic indiduals, this cohort may have a sense that they have reduced resources and capacity to make healthy lifestlye changes, even if the desire for change is present. Alternatively, it may be that circumstances dictate that other more pressing issues take greater preceidence over health, particulaly when “socioeconomically disadvantaged groups perceive price as a barrier to achieving a healthy diet” [3].

The physical environment also influences eating patterns in communities. In geographically isolated areas, where communities have reduced access to large supermarkets, people tend to use smaller convenience-type stores to gain access to food. In these stores, energy-dense foods are often cheaper than fruit, vegetables, and other healthy foods; therefore, purchasing power can lead to increased financial access to energy dense foods, particularly for those with lower income. Those among low- to middle-socioeconomic groups have also been shown to purchase and prepare more fast foods and ready-made meals due to a perceived lack of time to prepare homemade meals [3,4].

Overlaying the individual and physical environment levels is the social context in which individuals live. Eating may be viewed as a way for people to reconnect daily, celebrate events, or bring individuals together [4,7]. As a social process, the consumption of food is influenced by others within the group, where certain behaviours are accepted, promoted, or modelled [4]. This is also evident among low socioeconomic individuals who also have a propensity to value traditions, practices, and beliefs concerning food consumption that they were brought up with as children [4,7]. As such, food consumption among children is a result of parents’ food behaviours relating to purchasing, portion size, type, and eating patterns [4,7]. For example, parents’ intake of fruits and vegetables has a positive impact on the fruit and vegetables that their children consume. Children then carry these same behaviours and habits into adulthood [7]. 

In addition to modelling, the use of food, such as sweets or fast food, as a reward is a powerful motivator for children; however, as a reward, the desire for the specific food is increased and feelings associated with the specific food can perpetuate [7]. Similarly, negative association with healthy foods can be entrenched when rewards of preferential food are used.

It is within this context of individual and socioeconomic factors that this paper aims to examine the association between various foods consumed and eating patterns of children. Specifically, the paper seeks to determine if there are differences in food consumption between health-care and non-health-care card recipients. For this study, health-care card status was used as a proxy measure or indicator of income. 

A health-care card is an Australian Government entitlement scheme for those who earn a low income or who meet certain eligibility requirements, such as child or carer responsibilities. There are various health-care card types. The eligibility requirements for each type are often based on an assessment of age, income, and individual or family situation. For example, to be eligible for a ‘low income’ health-care card, a single parent with one dependent child would need to be earning less than $919 a week and parents with one child would need to be earning less than $953 a week combined. The health-care card entitles low-income recipients certain concessions for health care and pharmaceutical expenses including additional concessions on energy and electricity, public transport, and local government taxes [8].

## 2. Materials and Methods

### 2.1. Research Instrument

The “survey on eating patterns of children” was designed by a research team of dietitians, health promotion workers, a local government community wellbeing officer, and an academic. Development of the tool and detail of the tool has been described earlier [9]. The survey examined demographics of child and parent/carer, fruit and vegetable consumption of the child and additional factors that influence eating patterns, which has been published in detail previously [9]. 

This paper examines the correlations between children’s daily fruit and vegetable servings with servings of other foods and drinks. In addition, this paper explores differences in daily consumption of food and drinks between the children whose parents were health-care card recipients and those who were not.

Parents were asked about their child’s consumption of various food and drinks on a typical day, with descriptor amounts given for a serving size of each, in addition to a colour photograph of an example serving size [10]. Foods were listed separately as categories. Vegetables were described as fresh, frozen, cooked, or raw, and fruit as fresh, dried, or tinned. Snack examples were given as chips, cheezels, and muesli bars. Confectionary was defined as lollies and chocolate. Cake, doughnuts, sweet biscuits, and muffins were listed separately to the other snack examples. Drink examples were listed as water, plain milk, flavoured milk, fruit juice, soft drink, and cordial (un-carbonated sugar-sweetened beverage), again with a descriptor of serving size. A six point serving scale was provided, and included serving values such as 0, 1, 2, 3, 4, and 5 or more, or “I don’t know”. A copy of the survey is available from the corresponding author. 

Ethical approval for the study was granted by the Goulburn Valley Health Human Research Ethics Committee (EC00220) and the Department of Education and Early Childhood Development (2013_002125).

### 2.2. Data Collection

The study population was primary students in Grades 1 or 3 (aged 5 to 11 years) who attended one of the 41 primary schools that chose to participate across the three Local Government Areas of Greater Shepparton, Moira, and Strathbogie Shires in rural or regional North East Victoria. No other inclusion or exclusion criteria were set, but the survey was only provided in English. Surveys were distributed by teachers to the students to take home for their parent/carer to complete within a two-week period and be returned to the school for collection by the research team [9,10]. 

### 2.3. Data Analysis

Data were analysed using SPSS version 21 (IMB, Chicago, IL, USA). Descriptive statistics were generated for the demographic data and were used to characterise responses to questionnaire items [11], while inferential statistics examined group comparisons. These were undertaken using non-parametric tests such as Spearman’s rank-order correlation (ρ), Pearson’s Chi-Square tests, and Mann-Whitney U tests, as the data, even after being transformed, violated a number of parametric assumptions. Spearman’s rank-order correlation was used as the survey elicited information using a six point serving scale that was ordinal rather than continuous level data, where a small correlation was considered at 0.2 [12,13]. Significance was initially two-tailed *p* ≤ 0.05. However, this *p* value was adjusted using the Dunn-Šidák correction to account for the possibility of a Type I error rate resulting from multiple comparisons, that is, an increased family-wise error rate. We chose the Dunn-Šidák method over the more common Bonferroni correction because the latter can be overly conservative. That being said, there was a negligible difference in the resulting *p* values for the two methods in our case. We denote the Dunn-Šidák-adjusted *p*-value as *p*DS.

## 3. Results

The sample consisted of 550 respondents (28.5% response rate) from the City of Greater Shepparton (*n* = 258), Moira Shire (*n* = 233) and Strathbogie Shire (*n* = 59). Parent or carer respondents ranged in age from 23 to 73 with a mean age of 39.2 years (SD = 6.1). Education levels varied among respondents; 9.0% (*n* = 48) completed primary school, 24.6% (*n* = 131) completed secondary school, 61.9% (*n* = 330) completed tertiary education, and the remaining 4.5% (*n* = 24) completed other forms of education, such as vocation education. In addition, 32.5% of respondents (*n* = 174) indicated they were health-care card recipients, and reflective of the percentage of health-care card recipients across the three local government areas [14]. It must be noted that 14.1% (*n* = 78) of respondents were single parent/carer families, and 31.0% (*n* = 24) of these single parent/carer households were health-care card recipients and had a higher percentage of male children, as outlined in Table 1.

### 3.1. Overall Correlation between Food and Drink and between Health-Care and Non-Health-Care Card Households

When examining the overall cohort for the association that exists between fruit and vegetable servings and other foods, it was noted that there is a small positive correlation with fruit servings and consumption of water. In addition, there was a small negative correlation between vegetables and consumption of soft drinks (Table 2). When specifically examining the consumption of soft drinks, it was noted that there is a small negative correlation with the consumption of water and milk, while there is a medium positive correlation with sweets and a small positive correlation consumption of snacks, cordial, and fruit juice.

When examining the differences between health-care and non-health-care card recipients in terms of the types of foods that were consumed, both groups had correlations that were significant. For example, among those with a health-care card, the correlation between the number of fruit servings and the number of vegetable servings was smaller (ρ = 0.204, *p* < 0.009) than those who did not have a health-care card (ρ = 0.260, *p* < 0.001). Conversely, among those with a health-care card, the correlation between eating less healthy food (e.g., confectionary, cake, and snacks) fruits and vegetable servings was larger than among those who did not have a health-care card. 

In some cases, the correlation between soft drink and confectionary servings was almost one-and-a-half times larger (ρ = 0.620, *p* < 0.001) among health-care card holders than those who did not have a health-care card (ρ = 0.449, *p* < 0.001). For example, the correlation between soft drink, snacks, and fruit juice was larger among those households who had a health-care card, (ρ = 0.276, *p* < 0.001) and (ρ = 0.330, *p* < 0.001) than those who did not (ρ = 0.190, *p* < 0.001) and (ρ = 0.182, *p* < 0.001), respectively.

### 3.2. Fruit and Vegetables Cost between Health-Care and Non-Health-Care Card Households

There was also a positive correlation between agreement with the statement that fruit and vegetables cost too much and the servings of confectionary and snacks that were consumed by children. However, when examining health-care card and non-health-care card holders, it was indicated that among health-care card holders, the perception that fruit and vegetables cost too much was associated with increased servings of confectionary and snacks, while the same correlation is not observed among non-health-care card holders (Table 3). 

### 3.3. Food and Drink Servings between Health-Care and Non-Health-Care Card Households

When examining the differences between health-care and non-health-care card recipients, it was noted that children from households who had a health-care card consumed fewer vegetable servings than those children from households without a health-care card. However, there was no significant difference when examining fruit servings between the household types. The perception of cost of fruit and vegetables was highlighted to be significant among those who were health-care card holders, as outlined in Table 4. 

Children of health-care card recipients consumed fewer servings of water. Results also showed that these children consume more servings of fruit juice. A Mann-Whitney U test revealed a significant difference between the consumption of fruit juice among households with a health-care card and those that did not have a health-care card (U = 22019, z = −3.30, *p* < 0.001, ρ = 0.14).

Similarly, greater consumption of sweetened flavoured drinks and soft drink occurred among children from households that were health-care card recipients. A Mann-Whitney U test revealed a significant difference between the consumption of sweetened flavoured drinks (U = 21412, z = −4.65, *p* < 0.001, r = 0.21, and soft drink, U = 21701, z = −4.40, *p* < 0.001, ρ = 0.19) among households with a health-care card and those that did not have a health-care card.

## 4. Discussion

The main findings were that children who consumed more fruit and vegetables tended to have a higher intake of healthy drinks (plain milk and water) as well as a lower intake of unhealthy snacks and drinks (cake, sweet biscuits, fruit juice, and sugar sweetened drinks). However, respondents who perceived that fruit and vegetables cost too much reported greater consumption of unhealthy snacks and sugar-sweetened beverages. Ascertaining consumption of cordial and soft drinks was an important aspect of this study, as sugar-sweetened beverages have been shown to serve as a good proxy measure of consumption of other unhealthy foods [15]. This finding was more prominent in households that had a health-care card, where children consumed fewer vegetables, less water, and more fruit juice than those who do not have a health-care card. 

These findings resonate with current research, where lower-income households do not consume adequate fruit and vegetables and have a greater tendency to purchase and consume energy-dense foods, and children who consumed sweetened beverages also consumed unhealthy snack foods [2,16,17]. It has been suggested these behaviours are centred on the contributing factors such as poor nutritional knowledge, lack of time or confidence in meal planning and preparation, and the perceived high cost of healthier foods [18]. In addition, when examining regional, rural, and remote contexts, other geographical factors may also contribute to this phenomenon. These factors include distance to purchase food, the availability of public transport, the short shelf-life of fresh food, increased transport costs being passed on to consumers, and rural consumers previously experiencing food shortages [5]. 

However, beyond healthy food access and costs being inhibitory, other studies found that food choice and food consumption behaviours were also influenced by lower-income earners having a relatively low regard for food and the associated health issues, leading to food consumption being less consistent with dietary guideline recommendations [3,19]. Willis and colleagues [20] suggest that individuals and families use “hierarchies” of need and risk, which affect where purchasing nutritious food and health is placed in proportion to other items that are considered more important [21]. This reflects Bourdieu’s suggested notion of the “distances from necessity” [22], where individuals with lower incomes eat what is most filling and affordable, while food consumption among those with higher incomes is more nutritious, and they have a greater focus on preparation, presentation service, and entertaining guests. This may explain why popular food and lifestyle programs, such as Master Chef or Gourmet Farmer, are more popular among higher-income viewers, where cooking is considered fun rather than work, and these individuals can vicariously consume and critique food as armchair experts [23,24,25]. Distance from necessity may also indicate why nutritional interventions that promote healthy food consumption are less successful among those that who may be more socioeconomically disadvantaged [17,26]. 

Engel’s [27] law of consumption states that, as income rises, the proportion of money used to purchase food falls; even when food expenditure rises, the percentage of income used to purchase food is much less. However, Bourdieu [22] suggests that, when social conditions, such as high or low income, are experienced in early childhood, there is a propensity for these experiences to shape views of food, influence food consumption behaviours, and even tastes into adulthood. It is through these familial or social-cultural food practices where social order, ideologies, identity, and day-to-day practices are developed and maintained. Even in families, children are not passive recipients but have certain influences on familial food consumption patterns and behaviours [20,28]. Similarly, food consumption behaviours of a family are also influenced and perpetuated by the wider network of social relationships and social divisions or classes that exist in the community.

Despite these factors, individuals and groups may still be influenced by socio-cultural norms, there is the capacity to alter or shape food consumption behaviours, and it is suggested to be reflective of gaining a higher socioeconomic status or moving up the “social ladder” [20]. However, moving from the lowest socioeconomic group to the highest does not always ensure a change in food consumption behaviour. For example, it has been indicated that, even when controlling for income, food preferences and consumption behaviours are intergenerational and persist among children into adulthood [29]. This reiterates that food consumption, meaning and behaviours are social practices that develop, but also sustain self and family identities, social structures, and relationships [30].

### 4.1. Implications for Policy

It has been well established that the causes of unhealthy eating patterns and the resulting population weight gain are complex and inter-related [31]. The Foresight obesity systems map identifies thematic clusters of variables affecting energy balance ranging from individual through to societal and environmental factors, and may assist in the development of a more sophisticated and integrated policy approaches [32]. However, at the community level, community-based system dynamics has been identified as a promising health promotion approach that aims to address the complexity of unhealthy eating patterns. Using a participatory method, known as group model building, this approach seeks to engage local communities in developing real and meaningful solutions to address unhealthy eating behaviours [33,34]. Due to the differences observed between high- and low-income households in relation to eating patterns, a community-based initiative that examines the whole food system within a local context may provide opportunities for developing a better understanding of the reasons behind food and beverage consumption patterns within this target group. 

Interventions must also have an intergenerational focus, where there is greater recognition that the changing patterns and behaviours of food consumption occur over a much longer time, or even generations, rather than fleeting and condensed interventions, which have limited sustainability. Research [17,35] has suggested that it begins at the primary school level; however, these changes may not be seen for some time. This requires longitudinal research that evaluates healthy eating systems-based interventions specifically addressing the needs and barriers faced by socioeconomically disadvantaged individuals.

### 4.2. Limitations

The limitations of the study relate to respondents self-selecting to participate in the survey and the season in which the survey was completed. Those who did not respond to the survey were either parents/guardians who did not receive the survey from their child, or who chose not to participate in the study. Those who did not respond were not asked the reason for non-response, as the researchers did not directly recruit participants. It is recognised the non-response creates a level of bias in the results. Presumably, non-response is more likely to come from socioeconomically disadvantaged backgrounds and the survey was completed at a time when fresh fruits and vegetables were more abundant. Consequently, our findings are likely to underestimate the true magnitude of low fruit and vegetable intake and the consumption of unhealthy snacks and sugar-sweetened beverages. 

## 5. Conclusions

This study provides vital information about childhood fruit and vegetable consumption from one region in Victoria, enabling health promotion interventions specific to the populations included in the study. The results suggest that low-income families in this region should be considered as a sub-group in an effort to reduce sweetened beverage and unhealthy snack consumption. Furthermore, health promotion interventions must incorporate long-term, multi-level solutions, built on policy and community-based system dynamics theory. The survey developed and utilised for this study may be useful for other primary-care service providers wishing to undertake a similar investigation.

## Figures and Tables

**Table 1:** Characteristics of health-care and non-health-care card holder survey respondents.

Variables	Health-Care Card *n* (%) 174 (32.5%)	Non-Health-Care Card *n* (%) 376 (67.5%)
**Parent (*n* = 334)**		
− Father	8 (9.0%)	12 (5.0%)
− Mother	83 (89.0%)	228 (94.0%)
− Grandparent	2 (2.0%)	1 (1.0%)
**Parent/carer age groups (*n* = 531)**		
− 20–39 years	95 (55.0%)	175 (49.0%)
− 40–59 years	75 (44.0%)	183 (50.8%)
− 60 years and over	2 (1.0%)	1 (0.2%)
**Currently living (*n* = 535)**		
− Greater Shepparton Municipal	89 (35.0%)	164 (65.0%)
− Moira Shire	71 (32.0%)	152 (68.0%)
− Strathbogie Shire	14 (14.0%)	45 (76.0%)
**Education (*n* = 526)**		
− Completed primary	28 (16.0%)	20 (5.0%)
− Completed secondary	46 (27.0%)	84 (24.0%)
− Completed tertiary	89 (53.0%)	235 (66.0%)
− Other	6 (4.0%)	18 (5.0%)
**Household size (*n* = 531)**		
− Single parent/carer household	53 (31.0%)	10 (3.0%)
− Two parent/carer household	119 (59.0%)	349 (97.0%)
**Child sex (*n* = 533)**		
− Male	97 (56.0%)	166 (46.0%)
− Female	76 (44.0%)	194 (54.0%)
**Child age groups (*n* = 535)**		
− 5–6 years	26 (15.0%)	29 (8.0%)
− 7–8 years	85 (49.0%)	208 (58.0%)
− 9–11 years	63 (36.0%)	124 (34.0%)

**Table 2:** Spearman’s rank correlation between food and drink servings.

Food and Drink Consumed	Rho (ρ), Significance	Vegetables	Fruit	Water	Plain Milk	Snacks	Sweets	Cake	Fruit Juice	Soft Drink
Fruit	ρ	0.251 *	-	-	-	-	-	-	-	-
	Sig.	(0.001)								
Water	ρ	0.179 *	0.171 *	-	-	-	-	-	-	-
	Sig.	(0.001)	(0.001)							
Plain milk	ρ	0.151 *	0.115	0.102	-	-	-	-	-	-
	Sig.	(0.001)	(0.009)	(0.020)						
Snacks	ρ	−0.011	−0.035	−0.031	−0.068	-	-	-	-	-
	Sig.	(0.796)	(0.432)	(0.475)	(0.123)					
Sweets	ρ	−0.019	−0.047	−0.072	0.024	0.344 *	-	-	-	-
	Sig.	(0.674)	(0.299)	(0.109)	(0.600)	(0.001)				
Cakes	ρ	−0.089	0.002	−0.058	0.012	0.093	0.120	-	-	-
	Sig.	(0.042)	(0.971)	(0.185)	(0.778)	(0.034)	(0.007)			
Fruit juice	ρ	−0.129	−0.008	−0.111	−0.025	0.149 *	0.227 *	0.094	-	-
	Sig.	(0.004)	(0.853)	(0.014)	(0.585)	(0.001)	(0.001)	(0.036)		
Soft drink	ρ	−0.169 *	−0.142	−0.190 *	−0.093	0.225 *	0.419 *	0.127	0.273 *	-
	Sig.	(0.001)	(0.002)	(0.001)	(0.038)	(0.001)	(0.001)	(0.005)	(0.001)	
Cordial	ρ	−0.107	−0.064	−0.211 *	−0.059	0.225 *	0.201 *	0.056	0.172 *	0.233 *
	Sig.	(0.016)	(0.152)	(0.001)	(0.184)	(0.001)	(0.001)	(0.211)	(0.001)	(0.001)

* *p*_DS_ ≤ 0.0011 (2-tailed).

**Table 3:** Correlation between fruit and vegetables costing too much and other foods.

Participant Type	Fruit and Vegetable Cost	Rho (ρ), Significance	Snacks	Sweets	Cake	Fruit Juice	Soft Drink	Cordial
Health-care card	Vegetables cost	ρ	0.232 *	0.260 *	0.085	0.153	0.102	−0.054
		Sig.	(0.002)	(0.001)	(0.278)	(0.056)	(0.205)	(0.500)
	Fruit cost	ρ	0.283 *	0.269 *	0.141	0.206 *	0.149	−0.049
		Sig.	(0.001)	(0.001)	(0.073)	(0.009)	(0.064)	(0.537)
Non-health-care card	Vegetables cost	ρ	0.110 *	0.124 *	0.102	0.035	−0.028	0.096
		Sig.	(0.042)	(0.025)	(0.059)	(0.532)	(0.613)	(0.084)
	Fruit cost	ρ	0.078	0.054	0.137 *	0.019	−0.033	−0.005
		Sig.	(0.153)	(0.334)	(0.011)	(0.735)	(0.552)	(0.934)

* *p*_DS_ ≤ 0.0021 (2-tailed).

**Table 4:** Differences in food consumption of health-care and non-health-care card recipients.

Food and Drink Consumed According to Participant Type	*n*	Mean (SD)	Test (df) Statistic	*p*
Fruit consumption				
Health-care card recipient	167	2.06 (1.25)	χ^2^ (5, 519) = 6.69	0.245
Non-health-care card recipient	352	2.21 (1.00)		
Fruit costs too much				
Health-care card recipient	170	1.45 (0.55)	χ^2^ (2, 521) = 9.68	0.007
Non-health-care card recipient	351	1.32 (0.46)		
Vegetable consumption				
Health-care card recipient	169	1.61 (1.14)	χ^2^ (5, 523) = 23.12	<0.001 *
Non-health-care card recipient	354	2.02 (0.99)		
Vegetables cost too much				
Health-care card recipient	171	1.39 (0.54)	χ^2^ (2, 523) = 6.94	0.027
Non-health-care card recipient	352	1.30 (0.45)		
Water consumption				
Health-care card recipient	170	4.43 (1.60)	χ^2^ (7, 525) = 15.79	0.016
Non-health-care card recipient	355	4.77 (1.26)		
Cordial consumption				
Health-care card recipient	163	0.68 (0.97)	χ^2^ (7, 498) = 27.91	<0.001 *
Non-health-care card recipient	335	0.51 (0.95)		
Fruit juice consumption				
Health-care card recipient	159	0.97 (1.54)	χ^2^ (6, 489) = 25.30	<0.001 *
Non-health-care card recipient	330	0.41 (0.62)		
Soft drink consumption				
Health-care card recipient	158	0.45 (0.79)	χ^2^ (5, 488) = 22.71	<0.001 *
Non-health-care card recipient	330	0.23 (0.61)		

* *p* < 0.001 (2-tailed).
